# Plasma miR-193b-3p Is Elevated in Type 2 Diabetes and Could Impair Glucose Metabolism

**DOI:** 10.3389/fendo.2022.814347

**Published:** 2022-05-27

**Authors:** Hua Hu, Meng Zhao, Zhaoyang Li, Hongli Nie, Jia He, Zhuo Chen, Jing Yuan, Huan Guo, Xiaomin Zhang, Handong Yang, Tangchun Wu, Meian He

**Affiliations:** ^1^ Department of Occupational and Environmental Health and State Key Laboratory of Environmental Health for Incubating, School of Public Health, Huazhong University of Science and Technology, Wuhan, China; ^2^ Dongfeng Central Hospital, Dongfeng Motor Corporation and Hubei University of Medicine, Shiyan, China

**Keywords:** type 2 diabetes, microRNA, proteomics, glucose metabolism, case-control study

## Abstract

**Objective:**

To explore differentially expressed miRNAs in type 2 diabetes and their potential cellular functions.

**Methods:**

We screened plasma miRNAs by miRNA array analysis and validated them by TaqMan real-time PCR in 113 newly diagnosed, untreated type 2 diabetes cases and 113 healthy controls. Low-abundance plasma proteins encoded by miR-193b-3p target genes were explored in this study population. We further investigated the potential cellular functions of the differentially expressed miRNAs in HepG2 cells.

**Results:**

miR-193b-3p was differentially expressed in type 2 diabetes cases compared to healthy controls (fold change = 2.01, *P* = 0.006). Plasma levels of triosephosphate isomerase (TPI1, a protein involved in the glycolytic pathway) decreased in type 2 diabetes cases (fold change = 1.37, *P* = 0.002). The effect of miR-193b-3p on TPI1 was verified by transfection of miR-193b-3p into HepG2 cells. miR-193b-3p inhibited the expression of YWHAZ/14-3-3ζ in the PI3K-AKT pathway, subsequently altering the expression of FOXO1 and PCK1. After transfection, cells were incubated in glucose-free medium for another 4 h. Glucose levels in medium from cells with elevated miR-193b-3p levels were significantly higher than those in medium from negative control cells (*P* = 0.016). In addition, elevated miR-193b-3p reduced glucose uptake by inhibiting insulin receptor (IR) and GLUT2 expression.

**Conclusion:**

Plasma miR-193b-3p levels increased in type 2 diabetes cases, and TPI1 levels decreased in both plasma and HepG2 cells with increased miR-193b-3p levels, while extracellular lactate levels did not significantly changed. Moreover, miR-193b-3p may affect glucose metabolism by directly targeting YWHAZ/14-3-3ζ and upregulating the transcription factor FOXO1 downstream of the PI3K-AKT pathway.

## Introduction

Type 2 diabetes has been a growing global problem in last decades. Its main pathological mechanisms include insulin resistance in muscle, adipose, and liver tissues combined with dysfunction and subsequent failure of insulin-producing pancreatic beta cells (1–3). The global diabetes prevalence in 2019 is estimated to be 9.3% (463 million people), rising to 10.2% (578 million) by 2030 and 10.9% (700 million) by 2045. China has 116.4 million people with diabetes, causing a heavy medical and economic burden (4)..

MicroRNA (miRNA) molecules are short non-coding RNAs that mediate RNA silencing and post-transcriptional regulation of gene expression, negatively regulate the abundance of specific proteins, and then control numerous cellular and biological processes including metabolism (5). Accumulating evidence also suggests that miRNAs play an important role in cellular metabolic regulation (e.g., let-7 family), adipocyte differentiation (e.g., miR-133a), pancreatic development (e.g., miR-375, miR-26a-5p), and insulin biosynthesis, secretion, and signaling (e.g., miR-375, miR-7) (6, 7). For the cellular glucose metabolism, miRNAs play a pivotal role by targeting the key rate-limiting enzymes of relevant pathways to fine-tune control of metabolic homeostasis. Aberrant expression of these miRNAs can result in an over or under expression of key enzymes, such as phosphoenolpyruvate carboxykinase (PEPCK) and glucose-6-phosphatase (G6PC), contributing to the etiology of diabetes (8). Dysfunction in multiple tissues that control glucose homeostasis and insulin sensitivity often occurs years before diagnosis (9, 10). Diabetes is generally diagnosed based on elevated plasma glucose level which does not distinguish stage progression of diabetes (8). Recent studies have shown that miRNAs can be detected in circulating blood and can be future biomarkers for diagnosis of diabetes states (11). Plasma miRNAs, such as miR-122, have been shown to be differentially expressed at the progressive glycemic impairment stage (12), and miR-144-3p was found elevated in newly diagnosed diabetes (13). Previous studies explored the cellular functions of miRNAs or circulating miRNAs separately, and few of them have delved into the potential function of circulating miRNAs (14–18).

Proteins are important molecules in cellular functions. With the development of mass spectrometry proteomic approaches, studies have shown that low-abundance plasma proteins, such as adiponectin and resistin (19–21), are associated with diabetes risk (22, 23). Subsequent studies have also shown that these low-abundance proteins are involved in the development and progression of diabetes and are direct effector molecules in tissue or cellular dysfunction (24–26). miR-375 has been found to play an important role in the development and progression of diabetes by targeting messenger RNA (mRNA) transcripts and regulating protein expression (6, 7, 27, 28). However, most previous studies have explored the differences in plasma miRNA levels and plasma protein levels separately, and few have examined the differences in plasma miRNA and protein levels in the same study population or investigated their potential associations and underlying biological mechanisms in the development of type 2 diabetes.

Therefore, in the present study, we first explored the plasma miRNA profiles in 113 pairs of age- and sex-matched newly diagnosed, untreated diabetes cases and controls. Second, we examined the plasma proteomic profiles in the same study population and performed further bioinformatic analysis of miRNA target genes and signaling pathways enriched with the target genes. Finally, we verified the associations between miRNA and protein levels through *in vitro* experiments. We further explored the potential mechanism of the differential expression of plasma miRNAs in the development of type 2 diabetes through miRNA transfection and molecular biology experiments. The detailed workflow of this study is shown in **Supplementary Figure 1**.

## Materials and Methods

### Study Design and Population

The Dongfeng-Tongji (DFTJ) cohort was established in 2008 and enrolled 27,009 retired employees of Dongfeng Motor Corporation (DMC) who resided in Shiyan city, Hubei, China. Participants completed epidemiological questionnaires, provided blood samples, and participated in physical examinations at baseline enrollment in 2008. The participants were invited to participate in a follow-up examination in 2013, and the follow-up rate was 96.2% (n=25,978). Detailed information on the DFTJ cohort is described elsewhere (29). Participants were defined as having type 2 diabetes if they had a fasting plasma glucose level of ≥ 7.0mmol/L and/or a hemoglobin A1c (HbA1c) level of ≥ 6.5% (30). Individuals with type 2 diabetes who did not have cardiovascular disease or cancer and did not take any antidiabetic medication were selected as cases. Accordingly, a 1:1 age- and sex-matched population of individuals without diabetes, cancer, cardiovascular diseases, and medication use was selected as the control population. Finally, a total of 113 case-control pairs were enrolled in the present study. The detailed characteristics of the cases and controls are presented in **Table 1**. The 113 pairs of case-control samples were randomly divided into a screening group (n = 15 pairs) and a validation group (n = 98 pairs) to explore differentially expressed miRNAs. Considering the amount of plasma used for protein screening, twenty-five pairs of samples were randomly selected for protein screening, and an equal number of validation group samples were used for miRNA screening. The characteristics of the participants in the validation group are shown in **Supplementary Table 1**.

**Table 1:** Characteristics of the participants.

Variables	DM cases (n=113)	Controls (n=113)	*P*
Male, n (%)	53 (46.9)	53 (46.9)	1.0
Age, years	61.1 (7.1)	61.1 (7.1)	0.948
Smoking, n (%)			
Never	79 (70.5)	77 (68.1)	0.814
Current	21 (18.8)	25 (22.1)	
Ever	12 (10.7)	11 (9.7)	
Drinking, n (%)			
Never	80 (71.4)	73 (65.2)	0.525
Current	27 (24.1)	31 (27.7)	
Ever	5 (4.5)	8 (7.1)	
Physical activity, n (%)	102 (91.1)	100 (88.5)	1.0
BMI, kg/m^2^	25.3 (3.4)	22.9 (3.0)	<0.001
WHR	0.89 (0.06)	0.86 (0.06)	0.001
FPG, mmol/L	8.9 (2.4)	5 (0.4)	<0.001
HbA1c, %	6.5 (1.5)	5.3 (0.3)	<0.001
TG, mmol/L	2 (3.2)	1.3 (0.8)	0.022
LDLC, mmol/L	2.5 (0.9)	2.8 (0.8)	0.005
HDLC, mmol/L	1.5 (0.5)	1.6 (0.4)	0.477
TC, mmol/L	4.8 (1.5)	4.8 (1.1)	0.964
SBP, mmHg	137.7 (22.6)	135.8 (22.4)	0.522
DBP, mmHg	80.0 (13.1)	80.0 (13.0)	0.364
Neutrophil, 10^9^/L	3.7 (1.4)	3.3 (1.2)	0.058
Lymphocyte, 10^9^/L	2 (0.8)	1.7 (0.5)	0.003
Monocyte, 10^9^/L	0.4 (0.2)	0.3 (0.2)	0.046
Eosnophils, 10^9^/L	0.2 (0.2)	0.1 (0.1)	0.014
Basophil, 10^9^/L	0.2 (0.2)	0.1 (0.2)	0.297
WBC, 10^9^/L	6 (1.8)	5.4 (1.4)	0.008
RBC, 10^12^/L	4.5 (0.4)	4.5 (0.5)	0.205
PLT, 10^9^/L	183.3 (54.8)	196.9 (51.4)	0.056
Family history of diabetes, n (%)	9 (8.2)	8 (7.1)	0.685

WHR, waist-to-hip ratio.

All participants gave written informed consent. The study protocol was approved by the Ethics and Human Subject Committee of the School of Public Health, Tongji Medical College, Huazhong University of Science and Technology, and Dongfeng General Hospital, DMC.

### Plasma Sampling and RNA Isolation

Approximately 5 ml of venous blood was collected from each participant, placed in an EDTA-anticoagulant tube and centrifuged at 1000 × g for 10 min. Plasma was carefully transferred to an RNase-free tube and stored at −80°C until use. Prior to isolating miRNAs from plasma, we transferred the supernatant to a new tube after a brief centrifugation. Total RNA was isolated from plasma using a miRNeasy Serum/Plasma Kit (Qiagen) according to the manufacturer’s instructions. In total, 200 μl of plasma was used for the entire miRNA extraction. Approximately 1.6 × 108 copies/µl of synthetic Caenorhabditis elegans cel-miR-39 (Qiagen) was added to the denatured plasma samples as an internal control for the validation study. RNA sample concentrations were quantified using a NanoDrop ND-1000 (Nanodrop, USA).

### MiRNA Microarray Analysis

A MiRCURY LNA™ MicroRNA Array (Exiqon: 7th generation) was used for initial microarray screening. RNA samples were labeled and hybridized according to Exiqon’s manual. Scanned images were imported into GenePix Pro 6.0 software (Axon) for grid alignment and data extraction. Replicate miRNAs were averaged and miRNAs with intensities of ≥ 30 in all samples were selected to calculate the normalization factor. Expression data were processed using median normalization. After normalization, miRNAs that were significantly differentially expressed between the two groups were identified based on the fold change and P values. Volcanic Plots were used to visually indicate miRNAs with significant differences. Clustering was performed to show distinguishable miRNA (Fold Change >= 1.5, *P*-value <= 0.05) expression profiling among samples. After applying the Benjamini-Hochberg false discovery rate (FDR) correction for multiple comparisons, a P value of < 0.05 was considered a statistically significant difference. The R Statistical Software (http://www.r-project.org/), the ggplot2 Package (31), and pheatmap Package (32) were also used for the analysis of Volcanic Plots and Clustering, respectively. The microarray data have been submitted to the Gene Expression Omnibus (GEO) database (GSE134998; https://www.ncbi.nlm.nih.gov/geo/query/acc.cgi?acc=GSE134998).

### Quantification of Plasma miRNA

miRNA validation was carried out using TaqMan real-time quantitative PCR (qPCR). Reverse transcription (RT) was performed using miRNA-specific stem-loop RT primers and a MicroRNA RT Kit (Life Technologies) following the manufacturer’s instructions. The resulting cDNA was diluted and used immediately for qPCR or stored at −20°C until use. miRNA expression levels were measured by real-time qPCR in a ViiA ^7^ Real-Time instrument (Life Technologies) using TaqMan^®^ Universal Master Mix (Life Technologies). The miRNA expression levels were normalized to those of cel-miR-39 and determined by the equation 2^–ΔCt^, where ΔCt=cycle threshold (Ct) (miRNA) – Ct (cel-miR-39).

### Bioinformatic Analysis

The miRNA target sites in mRNA were predicted with miRDB (http://mirdb.org/), miRTarBase (http://mirtarbase.mbc.nctu.edu.tw/php/index.php) and TargetScan (http://www.targetscan.org/). Pathway enrichment analysis of the target genes was performed with the Kyoto Encyclopedia of Genes and Genomes (KEGG) database resource (http://www.genome.jp/kegg/). Bioinformatic analysis of the pathway enrichment results was performed with Database for Annotation, Visualization and Integrated Discovery (DAVID) tools (http://david.abcc.ncifcrf.gov/).

### Plasma Proteomics

The 113 case-control pairs were randomly divided into a preliminary screening dataset and a validation dataset. In the preliminary screening dataset, twenty-five plasma samples from each group were pooled into five samples, and isobaric tagging for relative and absolute quantification (iTRAQ)-based protein expression profiling was performed to identify proteins. The significantly and differentially expressed proteins were validated in the validation dataset by multiple reaction monitoring mass spectrometry (MRM-MS) with liquid chromatography-mass spectrometry (LC-MS), and synthetic peptides of beta-galactosidase (Sangon Biotech) were added to the denatured plasma samples as internal standard peptides.

### Cell Culture and miRNA Transfection

All *in vitro* experiments were performed in human hepatoma HepG2 cells procured from the National Infrastructure of Cell Line Resource, Beijing, China. The identity of all cell lines was confirmed by short tandem repeat profiling at the time of procurement in July 2018. Cells were maintained in high-glucose Dulbecco’s modified Eagle’s medium supplemented with 10% (v/v) heat-inactivated fetal bovine serum, 100 units/ml penicillin, and 100μg/ml streptomycin under conditions of 5% CO2/95% air at 37°C. HepG2 cells were transfected with either the mimic negative control (mimic_NC, 50nM) or the miRNA mimic (50nM) (RiboBio) with Lipofectamine 3000 and Plus Reagent (Invitrogen) according to the manufacturer’s instructions. When used, the miRNA inhibitor and inhibitor negative control were transfected at a dose of 100nM.

### RNA Isolation, qRT-PCR, and Western Blotting

After transfection for 48 h, cells were lysed in radio immunoprecipitation assay (RIPA) lysis buffer containing protease inhibitors. Total RNA was isolated from both cells and media using Invitrogen TRIzol (Life Technologies). Then, RNA (2μg) was reverse transcribed using random hexamers, and the expression levels of genes were measured with specific primers (**Supplementary Table 2**) and Applied Bio systems SYBR Green Master Mix (Life Technologies). Proteins were isolated from both cells and media for *in vitro* experiments by cold acetone sedimentation. Protein samples (30μg) were analyzed by Western blotting (primary antibodies are listed in **Supplementary Table 3**).

### Glucose Production Assay and Lactate Measurements

HepG2 cells were transfected with the miRNA mimic or inhibitor for 48 h. Prior to termination of culture, cells were incubated in 12h serum starvation conditions in DMEM without glucose plus L-glutamine (Cat No: 11966025, Gibco) and then were incubated 4h in the same medium supplemented with 10% FBS. Extracellular lactate levels were estimated by a lactate assay kit (Sigma-Aldrich), and glucose levels were estimated by the glucose oxidase method.

### Glucose Consumption and Intracellular Glycogen Content Measurements

After HepG2 cells were transfected with the miRNA mimic or inhibitor and cultured in high-glucose DMEM for 48 h, the median glucose levels were measured by the glucose oxidase method. Cell viability was assessed with a Cell Counting Kit-8 (CCK-8) assay (Dojindo Molecular Technologies). Glucose consumption was normalized by the CCK-8 assay. The anthrone-sulfuric acid colorimetric method was used to measure the intracellular glycogen content, which was normalized to the protein content (Pierce™ Rapid Gold BCA Protein Assay Kit, Life Technologies). To estimate the effect of insulin, after 24 h of transfection, cells were serum starved for 12 h and then incubated for 2 h with insulin (100nM).

### Statistical Analysis

Differences in clinical characteristics, plasma miRNA levels, and plasma protein levels between type 2 diabetes cases and controls were evaluated by chi-square test for categorical variables, by Student’s t test for normally distributed data or by Mann–Whitney U test for skewed data. Correlations between plasma miRNA profiles or protein expression levels (log transformed and normally distributed) and clinical measurements were evaluated by Pearson correlation analysis. Multivariate logistic regression models were used to calculate the odds ratios (ORs) and 95% confidence intervals (CIs). All bars in figures indicate the mean ± standard deviation (SD) values, and data were analyzed using ANOVA with a *post hoc* test.

## Results

### Characteristics of the Study Population

As shown in **Table 1**, BMI, waist-hip ratio (WHR), and levels of fasting plasma glucose (FPG), HbA1c, and triglyceride (TG) were significantly higher but the levels of low-density lipoprotein cholesterol (LDLC) were lower in type 2 diabetes cases than in controls. Compared with controls, type 2 diabetes cases had elevated white blood cell counts. Similarly, neutrophil count, lymphocyte count, and eosinophil count were significantly higher in type 2 diabetes cases than in controls.

### Plasma miRNA Profiles in Type 2 Diabetes Cases and Controls

**Figure 1:**
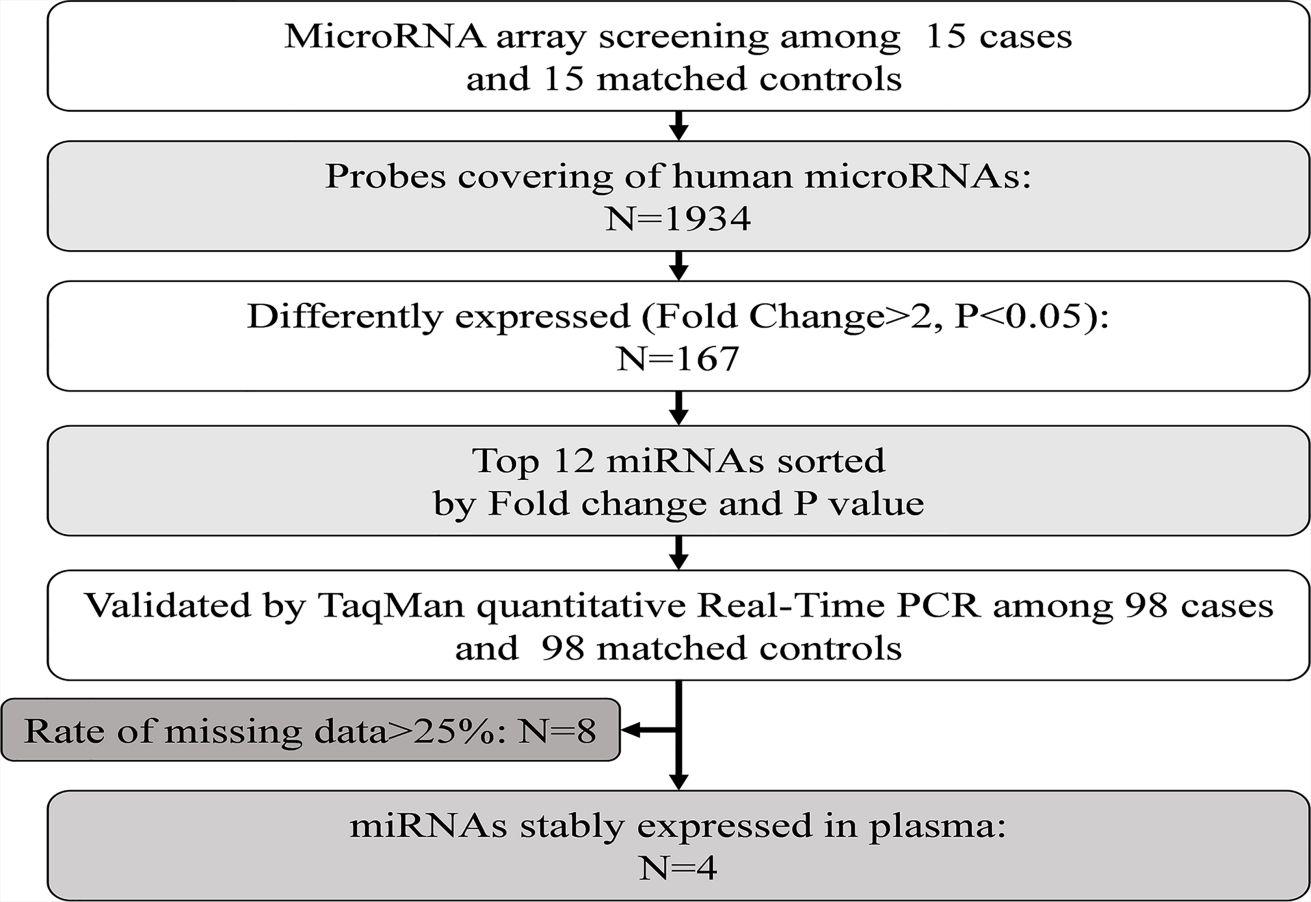
Study design for the plasma miRNA profile in type 2 diabetes cases and controls. PCR, polymerase chain reaction.

**Figure 2:**
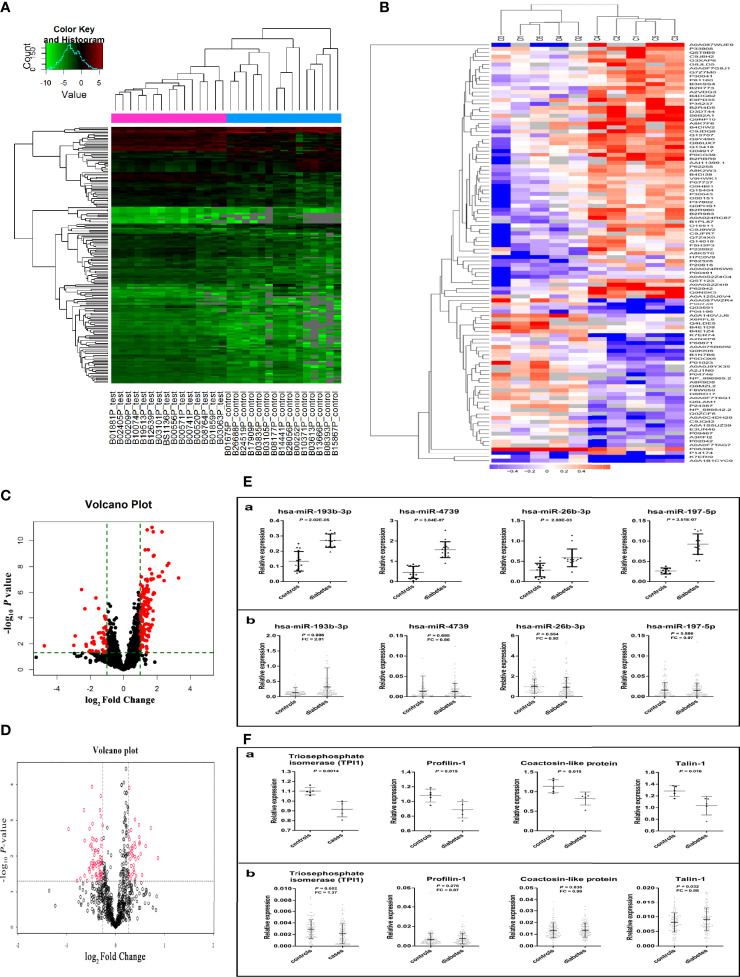
Results of miRNAs and protein screening and validation. **(A)** shows the results of cluster analysis of differently expressed miRNAs. Sample numbers with the test suffix are type 2 diabetes cases (like B01881P_test). Sample numbers with the control suffix are healthy controls (like B01675P_control). **(B)** shows the results of cluster analysis of differently expressed proteins. C1-C5: type 2 diabetes cases, D1-D5: healthy controls. **(C)** The volcano plot of miRNAs in array screening. **(D)** The volcano plot of proteins. (**E**. a) Differential expression of miRNA in plasma screened by miRNA microarray chip. (**E**. b) Differential expression of miRNA in plasma validated by Taqman PCR. (**F**. a) The different expression of 4 plasma proteins in preliminary screening group. (**F**. b) The different expression of 4 plasma proteins in validation group. (The figures were created by graphPad and R Statistical Software).

Plasma miRNA profiles in 15 case-control pairs were assessed by miRNA array screening (**Figure 1**). Among the 1934 detected miRNAs, 167 were differentially expressed between the two groups. The results of cluster analysis of differentially expressed miRNAs are shown in **Figure 2A**. We selected top 12 miRNAs (fold change and *P* values are listed in **Table 2**) for validation in a larger population (98 type 2 diabetes cases and 98 matched controls) using TaqMan real-time PCR, and the expression levels of 4 miRNAs were successfully measured (To improve the repeatability, we excluded 8 miRNAs for further analysis. Because the plasma levels of these miRNAs were below the instrument detection limit when we validated the 12 miRNAs by PCR.). The detailed expression profiles of miR-193b-3p, miR-26b-3p, miR-197-5p, and miR-4739 are shown in **Figure 2** **(E. b)**. As the results indicate, the plasma levels of miR-193b-3p were significantly elevated in type 2 diabetes cases compared with that in controls (fold change =2.01, *P* = 0.006).

**Table 2:** Differently expressed miRNAs selected to validation.

miRNAs	Fold change	P value	FDR	CV	
				Controls	Diabetes
miR-3591-5p	0.17	5.99E-07	4.22E-05	0.5	0.28
miR-122-3p	0.32	2.66E-06	1.00E-04	0.43	0.38
miR-193b-3p	2.02	2.45E-07	2.02E-05	0.48	0.16
miR-26b-3p	2.11	1.00E-04	2.00E-03	0.58	0.37
miR-300	2.44	5.06E-10	1.56E-07	0.52	0.12
miR-217	2.52	7.96E-07	5.30E-05	0.43	0.3
miR-3926	2.66	1.46E-11	1.38E-08	0.43	0.15
miR-641	2.68	1.31E-07	1.13E-05	0.51	0.28
miR-593-5p	3.13	1.04E-07	1.03E-05	0.61	0.27
miR-105-5p	3.22	3.52E-08	5.40E-06	0.65	0.28
miR-4739	3.45	1.59E-09	3.84E-07	0.65	0.25
miR-197-5p	3.53	1.35E-09	3.51E-07	0.28	0.27

CV, coefficient of variance, FDR, False Discovery Rate, FDR is calculated from Benjamini Hochberg FDR.

Multivariate logistic analysis suggested that plasma miR-193b-3p levels were significantly associated with elevated diabetes risk (OR: 2.11, 95% CI: 1.02-4.37) after adjustment for age, sex, BMI, smoking status, drinking status, and family history of diabetes. Further adjustment for total cholesterol (TC) levels and systolic blood pressure (SBP) slightly enhanced the association (OR: 2.28, 95% CI: 1.05-4.91). Similarly, after further adjustment for white blood cell count, red blood cell count, and platelet (PLT) count, the miR-193b-3p level was consistently associated with increased type 2 diabetes risk (OR: 2.25, 95% CI: 1.05-4.84) (**Table 3**). In addition, plasma miR-193b-3p levels were significantly related to TC, TG, FPG, HbA1c, and lymphocyte count (*P* < 0.01) (**Table 4**).

**Table 3:** Association of 4 miRNAs with T2D risk in validation population.

	Model 1		Model 2		Model 3	
	OR (95%CI)	*P*	OR (95%CI)	*P*	OR (95%CI)	*P*
miR-193b-3p	2.11 (1.02,4.37)	0.046	2.28 (1.05,4.91)	0.036	2.25 (1.05,4.84)	0.038
miR-4739	1.00 (0.70,1.44)	0.991	1.01 (0.70,1.44)	0.975	1.05 (0.73,1.52)	0.793
miR-26b-3p	0.80 (0.58,1.12)	0.194	0.81 (0.57,1.15)	0.237	0.87 (0.59,1.28)	0.471
miR-197-5p	0.92 (0.66,1.27)	0.602	0.94 (0.67,1.30)	0.689	0.96 (0.68,1.35)	0.824

Model 1: adjusted for sex, age, BMI, smoking status, drinking status, physical activities and family history of diabetes.

Model 2: adjusted for variables in model 1 and TC (total cholesterol) and SBP (systolic pressure).

Model 3: adjusted for variables in model 2 and WBC (white blood cell count), RBC (red blood cell count) and PLT count.

**Table 4:** Pearson correlation coefficients of plasma miRNAs correlation to clinical biochemical measurements and blood cell counts was analyzed in healthy participants (n=49).

	miR-193b-3p	miR-4739	miR-26b-3p	miR-197-5p
**Clinical biochemical measurements**				
SBP	0.101	0.065	0.250**	0.207**
DBP	0.083	-0.034	0.141	0.049
TG	0.283**	-0.021	0.126	0.12
LDLC	0.008	-0.005	0.228**	-0.035
HDLC	-0.118	-0.003	-0.101	-0.004
CHOL	0.185*	-0.033	0.177*	-0.03
FPG	0.274**	0.009	-0.133	-0.098
HbA1c	0.237**	-0.033	0.218*	-0.032
**Blood cell counts**				
Neutrophil	0.023	-0.088	0.254**	0.019
Lymphocyte	0.277**	0.067	0.11	0.045
Monocyte	0.105	0.004	0.031	0.114
Eosnophils	-0.054	0.021	0.047	0.075
Basophil	-0.045	-0.042	-0.036	-0.029
RBC	0.012	-0.048	0.161*	0.057
PLT	-0.011	0.011	0.297**	0.036

* indicates correlations statistically significant: *P < 0.05, **P < 0.01.

### Target Genes of miR-193b-3p

Based on three commonly used target algorithm tools (TargetScan, miRDB, and miRTarBase), we performed pathway enrichment analysis on genes predicted by at least two of the tools. The PI3K-AKT signaling pathway was predicted with an FDR of < 0.05 (**Supplementary Figure 2**). Three genes including SOS Ras/Rho guanine nucleotide exchange factor 2 (SOS2), the GTPase KRas (KRAS) (33), and tyrosine 3-monooxygenase/tryptophan 5-monooxygenase activation protein zeta (YWHAZ) (34) were potential direct targets of miR-193b-3p in the PI3K-AKT signaling pathway and have been reported to be involved in cellular glucose metabolism. Therefore, we speculated that miR-193b-3p might regulate cellular glucose metabolism by directly targeting SOS2, YWHAZ, and KRAS.

### Plasma Protein Profiles in Type 2 Diabetes

In total, 815 proteins were detected in pooled plasma by iTRAQ. The differentially expressed proteins are presented in **Figure 2B**. The mRNAs encoding 35 of these proteins were predicted to be miR-193b-3p targets by three commonly used target algorithm tools (TargetScan, miRDB, and miRTarBase) (**Supplementary Figure 3**). Identified proteins encoded by target genes are listed in **Supplementary Table 4**. Differentially expressed proteins are shown in **Table 5**. Triosephosphate isomerase (TPI1, P60174), profilin-1 (PFN1, P07737), talin-1 (TLN1, Q9Y490), and coactosin-like protein (COTL1, Q14019) were selected for further validation in a larger population (fold change > 1.2, *P* < 0.05, and coverage of identified peptides > 50%). Proteins with low coverage of identified peptides, including O15511, P21333, and B3GN61 were not selected for validation (**Table 6**). The fold change values and *P* values of the other 28 proteins are shown in **Supplementary Table 5**. KIT and YWHAZ were predicted by three databases, but YWHAZ was not differentially expressed between the two groups, and the coverage of identified peptides for KIT was less than 50%.

**Table 5:** Differentially Expressed Proteins Identified by iTRAQ Analysis.

Accession	Description	Peptides Coverage	Case vs. Control	
			Fold change	*P* value
Q4LDE5	Sushi, von Willebrand factor type A, EGF and pentraxin domain-containing protein 1(SVEP1)	0.31	1.41	0.004
P60174	Triosephosphate isomerase 1 (TPI1)	61.85	0.83	0.001
P07737	Profilin-1 (PFN1)	73.57	0.82	0.015
Q9Y490	Talin-1 (TLN1)	53.56	0.81	0.016
Q7Z7M0	Multiple epidermal growth factor-like domains protein 8 (MEGF8)	6.12	0.8	0.001
Q14019	Coactosin-like protein (COTL1)	51.41	0.73	0.018
O15511	Actin-related protein 2/3 complex subunit 5 (ARPC5)	38.41	0.71	0.016

Accession was the number of proteins in Uniprot database.Peptides coverage: The number of amino acids in the peptide detected by mass spectrometry accounted for a proportion of the total number of amino acids in the protein.

**Table 6:** Differentially Expressed Proteins Identified by iTRAQ Analysis.

Accession	Description	Peptides Coverage	Case vs. Control	
			Fold change	*P* value
Q4LDE5	Sushi, von Willebrand factor type A, EGF and pentraxin domain-containing protein 1(SVEP1)	0.31	1.41	0.004
P60174	Triosephosphate isomerase 1 (TPI1)	61.85	0.83	0.001
P07737	Profilin-1 (PFN1)	73.57	0.82	0.015
Q9Y490	Talin-1 (TLN1)	53.56	0.81	0.016
Q7Z7M0	Multiple epidermal growth factor-like domains protein 8 (MEGF8)	6.12	0.8	0.001
Q14019	Coactosin-like protein (COTL1)	51.41	0.73	0.018
O15511	Actin-related protein 2/3 complex subunit 5 (ARPC5)	38.41	0.71	0.016

Accession was the number of proteins in Uniprot database.Peptides coverage: The number of amino acids in the peptide detected by mass spectrometry accounted for a proportion of the total number of amino acids in the protein.

Two peptides per protein were selected for relative quantification of protein levels in plasma from 98 controls and 98 type 2 diabetes cases. The Q1/Q3 transitions of target proteins and the internal standard protein (beta-galactosidase) are shown in **Table 7**. As shown in **Figure 2** **(F. b)**, plasma TPI1 levels significantly decreased (fold change = 1.37; *P* = 0.002) but talin-1 levels marginally but significantly increased (fold change = 1.13; *P* = 0.032) in type 2 diabetes cases. However, no significant difference was observed for plasma levels of profilin-1 and coactosin-like protein. A non-significant negative correlation between plasma miR-193b-3p and TPI1 was observed (**Supplementary Figure 4**).

**Table 7:** Q1/Q3 transitions of 4 target proteins selected for the MRM experiments.

Accession	Protein Name	Peptide Sequence	Q1/Q3 (m/z)	DP	CE
P00722	β-galactosidase	GDFQFNISR	542.3/489.3	70.6	28.4
			542.3/636.3	70.6	28.4
		VDEDQPFPAVPK	671.3/587.2	80.1	33
			671.3/755.4	80.1	33
P07737	Profilin-1	DSPSVWAAVPGK	607.3/913.4	75.4	30.7
			607.3/301.2	75.4	30.7
		TFVNITPAEVGVLVGK	822.5/968.6	91.1	38.5
			822.5/1069.6	91.1	38.5
P60174	Triosephosphate isomerase	SNVSDAVAQSTR	617.8/562.3	76.2	31.1
			617.8/934.5	76.2	31.1
		VVLAYEPVWAIGTGK	801.9/1057.6	89.6	37.7
			801.9/928.5	89.6	37.7
Q14019	Coactosin-like protein	EVVQNFAK	312.2/130	53.9	14.6
			312.2/147.1	53.9	14.6
		FALITWIGENVSGLQR	902.5/473.3	96.9	41.3
			902.5/959.5	96.9	41.3
Q9Y490	Talin-1	AVASAAAALVLK	542.8/914.6	70.7	28.4
			542.8/685.5	70.7	28.4
			362.2/147.1	57.5	17.3
			362.2/260.2	57.5	17.3
		GLAGAVSELLR	543.3/617.4	70.7	28.4
			543.3/716.4	70.7	28.4

Accession was the number of proteins in Uniprot database, Q1: parent ion, Q2: transition; DP=Declustering Pressure; CE=Collision Pressure.

**Figure 3:**
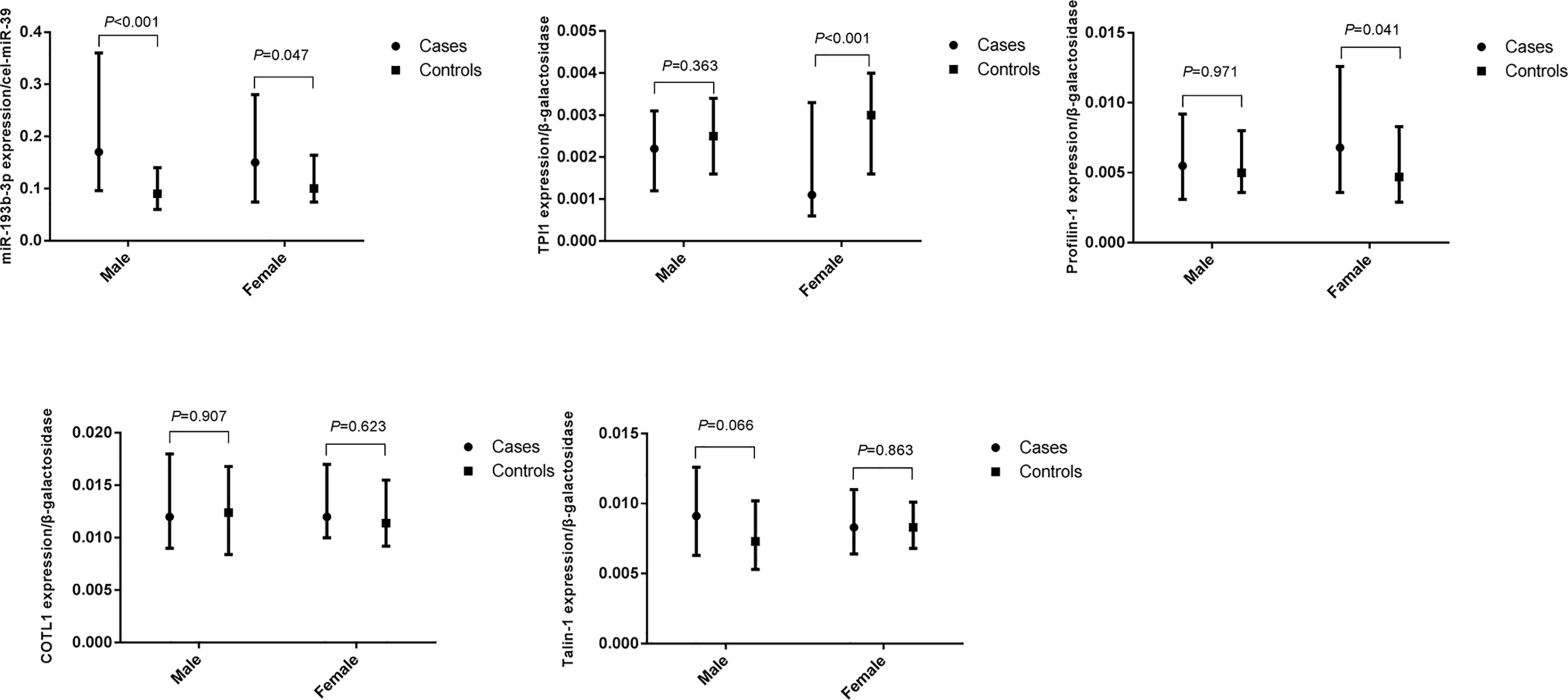
Differences of plasma miR-193b-3p and proteins between healthy controls and type 2 diabetes cases in groups according to gender. (The figures were created by graphPad).

We further explored the difference in miR-193b-3p and protein levels between type 2 diabetes cases and controls in males and females (**Figure 3**). No significant differences in plasma miR-193b-3p levels were observed between males and females, while TPI1 levels significantly reduced in females but not in males.

### Effects of miR-193b-3p on TPI1 Expression and Glycolysis in HepG2 Cells

**Figure 4:**
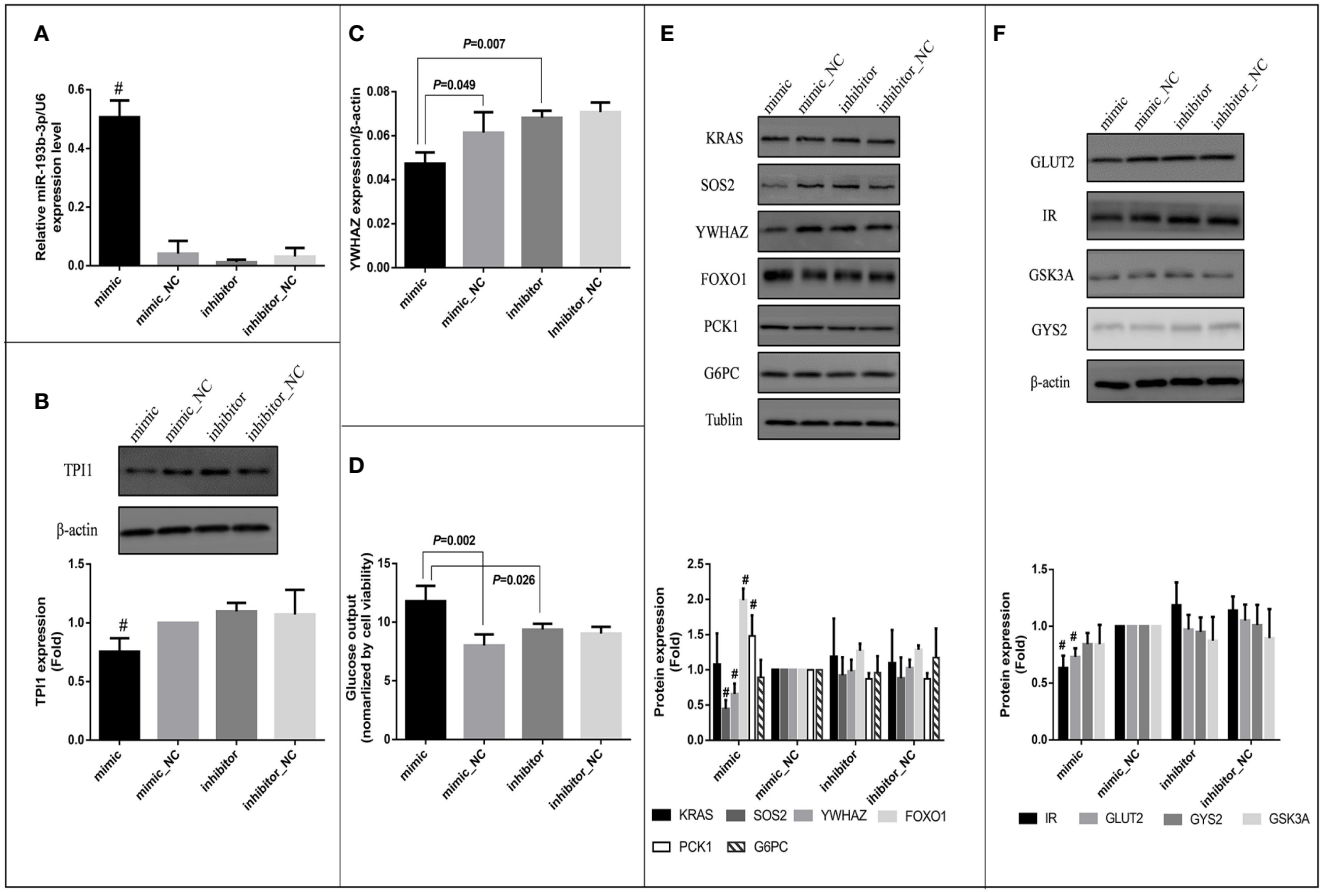
Effects of miR-193b-3p on glucose metabolism in HepG2 cells. Mimic: HepG2 cells transfected with miR-193b-3p mimic; mimic_NC: cells transfected with negative control of mimic; inhibitor: cells transfected with miR-193b-3p inhibitor; inhibitor_NC: cells transfected with negative control of inhibitor. **(A)** The expression level of miR-193b-3p after transfection; **(B)** Representative Western blot of TPI1 and β-actin after transfection. **(C)** The mRNA expression level of YWHAZ. **(D)** The level of extracellular glucose after transfection. **(E)** Representative Western blot of KRAS, SOS2, YWHAZ, FOXO1, PCK1, G6PC, and Tublin after transfection. **(F)** Representative Western blot of GLUT2, IR, GSK3A, GYS2, and β-actin after transfection. The bar graphs of figure **(B)**, **(E)** and **(F)** showed the fold change of protein levels (cells transfected with mimic negative control as reference group) quantified by Image Pro Plus, # indicates *P <*0.01 for protein level in mimic group compared with all the other groups. (The figures were created by graphPad).

Since TPI1 mRNA was predicted to be the direct target of miR-193b-3p, a miR-193b-3p mimic was transfected into HepG2 cells to evaluate the effects of miR-193b-3p on TPI1 gene expression. MiR-193b-3p was overexpressed in the mimic group compared with the mimic_NC group, inhibitor group, and inhibitor negative control group (**Figure 4A**). However, cell viability did not differ between the four groups (**Supplementary Figure 5A**). We further examined the mRNA and protein levels of TPI1 in transfected cells. The TPI1 protein level (**Figure 4B**) significantly decreased in mimic group compared with other groups (*P* < 0.01), while the TPI1 mRNA levels did not significantly differ between the mimic group and the negative control group (**Supplementary Figure 5B**). Since TPI1 is an important isomerase in the cellular glycolytic process (35), we further measured extracellular lactate levels. As shown in **Supplementary Figure 5C**, lactate levels did not significantly differ between the mimic group and other treatment groups.

### MiR-193b-3p Reduced YWHAZ and SOS2 Expression Levels, Subsequently Altering PCK1 Expression and Gluconeogenesis in HepG2 Cells

Bioinformatic analysis indicated that miR-193b-3p might directly target SOS2, YWHAZ, and KRAS and thus regulate cellular glucose metabolism. *In vitro*, mRNA levels of YWHAZ significantly decreased in the miR-193b-3p mimic group compared to miRNA mimic_NC (*P*=0.049) and miRNA inhibitor group (*P*=0.007) 48 h after miR-193b-3p mimic transfection (**Figure 4C**), while SOS2 and KRAS levels did not significantly change (**Supplementary Figures 5D, E**). Moreover, the protein levels of YWHAZ and SOS2 were significantly decreased in the miR-193b-3p mimic group (**Figure 4E**) compared to other groups (*P* < 0.01). YWHAZ has been shown to regulate the expression of FOXO1 (36, 37). In addition, as a transcription factor in the PI3K/AKT signaling pathway, FOXO1 has been shown to increase the expression of genes encoding proteins involved in gluconeogenesis, including phosphoenolpyruvate carboxykinase (PCK1) and glucose 6-phosphatase (G6PC) (38, 39). Therefore, we further measured the expression levels of FOXO1, PCK1, and G6PC. Although mRNA levels of FOXO1, PCK1, and G6PC did not significantly change (**Supplementary Figures 5F–H**), the protein levels of FOXO1 and PCK1 were significantly increased in the mimic group compared with other groups (**Figure 4E**, *P* < 0.01). As shown in **Figure 4D**, when cells were incubated in glucose-free medium for another 4 h after transfection, the glucose level in the medium of the mimic group was significantly increased compared to those in the medium of the other groups (mimic vs. mimic_NC: *P* =0.002; mimic vs. inhibitor: *P* = 0.026).

### MiR-193b-3p Altered Glucose Uptake in HepG2 Cells

Since SOS2 is an important upstream factor in the PI3K/AKT signaling pathway (40, 41), we further estimated the glucose consumption and intracellular glycogen content of the transfected cells after incubation in high-glucose DMEM for another 48 h. Both glucose consumption (*P* = 0.032) and the intracellular glycogen content (*P* = 0.009) were significantly decreased **(** **Supplementary Figures 5I, J**) in the miRNA overexpression group compared to miRNA knockout group (mimic vs. inhibitor), whereas the expression levels of glycogen synthase kinase (GSK3A) and glycogen synthase (GYS2) did not significantly change (**Supplementary Figures 5L, K**). Furthermore, we investigated factors affecting glucose uptake and found that the protein levels of insulin receptor (IR) and glucose transporter 2 (GLUT2) were significantly decreased in the miR-193b-3p mimic group compared to other groups (**Figure 4F**). To further explore the effects of miR-193b-3p on insulin-mediated glucose uptake, we estimated glucose consumption and the intracellular glycogen content 2 h after insulin stimulation. Both were significantly decreased in the mimic group compared to negative control group (*P* = 0.015 and 0.049, respectively) (**Supplementary Figures 5O, P**).

## Discussion

It has demonstrated that miRNAs play an important role in the glucose metabolism (8). MiRNAs in circulating blood could be potential biomarkers of diabetes (11). Based on a case-control study we found that plasma miR-193b-3p levels increased in newly diagnosed, untreated diabetes cases. The *in vitro* experiments indicated that elevated levels of miR-193b-3p in cells may impair glucose metabolism by inhibiting the expression of SOS2 and YWHAZ/14-3-3ζ in the PI3K-AKT pathway. These findings provided new evidence to the important role of miRNAs in the diabetes development.

Recent studies focused on delineating circulating miRNA profiles to find new disease biomarkers (9, 15). Previous studies showed that plasma levels of miR-122 (42), miR-126 (43, 44), and circulating exosomal miR-20b-5p (9) elevated in type 2 diabetes cases and may affect insulin function. Furthermore, a previous study showed that plasma miR-193b-3p levels significantly increased in a prediabetic population and glucose-intolerant mice (16). Moreover, circulating miR-193b-3p levels returned to baseline levels in glucose-intolerant mice receiving chronic exercise therapy intervention. In addition, polycystic ovary syndrome (PCOS) patients with impaired glucose metabolism had increased serum miR-193b-3p levels compared with PCOS patients with normal glucose tolerance (45). However, the potential mechanism of miR-193b-3p in cellular glucose metabolism has not been extensively explored. Limited evidence suggested that miR-193b-3p controls adiponectin production in human white adipose tissue (46), which is strongly and inversely associated with diabetes risk (25).

In the present study, plasma miR-193b-3p levels were related to TG, HbA1c, and FPG levels in healthy controls. In addition, SOS2, KRAS, and YWHAZ/14-3-3ζ in the PI3K-AKT signaling pathway were associated with glucose metabolism, as shown by miR-193b-3p target gene enrichment analysis (23, 34, 40). Currently, *in vitro* studies on glucose metabolism mainly focused on hepatogenic cells, most commonly human hepatocellular carcinoma HepG2 cells (47); therefore, we further explored the effect of miR-193b-3p on glucose metabolism in HepG2 cells.

YWHAZ/14-3-3ζ, a member of the 14-3-3 protein family, is a direct target of miR-193b-3p in MCF-7 cells (48) and can directly downregulate the expression of FOXO1, an important transcription factor downstream of the PI3K-AKT pathway (37, 49). In the present study, we validated the association of miR-193b-3p with YWHAZ at both mRNA and protein levels. Moreover, we found that as miR-193b-3p level increased, the protein levels of FOXO1 and PCK1 also elevated. Similarly, glucose output from the cells increased, consistent with previous findings indicating that FOXO1 increased the expression of genes encoding proteins involved in gluconeogenesis, including PCK1 and G6PC (38, 39). In summary, as shown in **Supplementary Figure 6**, miR-193b-3p can target YWHAZ/14-3-3ζ and subsequently upregulate transcription factor FOXO1 downstream of the PI3K-AKT pathway, which increases PCK1 expression, having a potential effect on enhancing gluconeogenesis. Further experiments are required to prove this hypothesis.

The present study also showed that the protein level of SOS2 decreased as the level of miR-193b-3p increased. SOS2 has been reported to be involved in positive regulation of Ras proteins (50) as an important upstream factor in the PI3K-AKT pathway and can cooperate with other factors to activate the PI3K-AKT pathway in (51). Therefore, SOS2 may not significantly affect the expression of AKT (a serine/threonine kinase), which can be activated by phosphatidylinositol 3-kinase (PI3K) and plays an important role in processes of glucose metabolism, including glycogen synthesis (49). Accordingly, the expression of GSK3A and GYS2 did not significantly change with elevated miR-193b-3p levels, which were directly affected by AKT (52).

In addition, decreased expression levels of IR and GLUT2 proteins were observed, but the mRNAs encoding these proteins were not predicted to be direct targets of miR-193b-3p in the miRNA-target databases. These reductions may be due to enhanced gluconeogenesis. Increased intracellular gluconeogenesis reduces the concentration gradient of glucose across the membrane, thereby decreasing GLUT2 expression and glucose uptake (53). In addition, the 14-3-3 protein has been reported to inhibit the expression of insulin receptor substrate 1 (IRS1), leading to insulin resistance (54), although the exact mechanism needs to be further explored.

We observed that as the miR-193b-3p levels increased, the TPI1 levels decreased in plasma and cells. Given that TPI1 is an enzyme involved in glycolysis without apparent tissue specificity (reference from Expression Atlas: https://www.ebi.ac.uk/gxa/home), we cannot attribute its plasma down regulation to a specific cell type yet. Based on the results of gene enrichment analysis and liver being one of the main organs involved in the regulation of glucose metabolism, we chose the hepatocytes to explore the potential function. For the potential mechanism of decreased level of TPI1 in HepG2, On the one hand, miR-193b-3p may directly target TPI1 mRNA through noncanonical binding mode, as previously mentioned (55). On the other hand, the central glycolytic enzyme TPI1 has been reported to play a key role in linking energy with redox metabolism during the stress response and in cancer, and the pyruvate kinase (PK) substrate phosphoenolpyruvate (PEP) can inhibit TPI1 activity in the feedback regulation system of glycolysis (35). In the present study, the protein levels of PCK1 increased in cells as miR-193b-3p levels elevated. Together with GTP, PCK1 catalyzes the formation of PEP from oxaloacetate and releases carbon dioxide and GDP (56). Thus, miR-193b-3p might indirectly affect cellular function of TPI1.

*In vitro* cell experiments, the expression of miR-193b-3p in the miRNA mimic group increased more than 5~10-fold compared to the mimic_NC group, while the expression of SOS2 and KRAS on RNA level did not significantly change. However, significant differences in protein expression levels and glucose output were observed. Previous studies indicated that miRNAs can negatively regulate gene expression by targeting specific mRNA transcripts and inducing their degradation or translational repression (39). According to the results of this study, and based on the transcripts analyzed, we suggest that miR-193b-3p inhibit the translation rather than inducing the degradation of target mRNAs encoding proteins including IR and GLUT2 that mediating glucose uptake. Our results illustrated the effects of miR-193b-3p overexpression on cellular function, however, the detailed effects of miR-193b-3p on protein and the effects of cytokines on downstream proteins and the potential mechanism needed to be further explored. Regarding the effect of miR-193b-3p on glucose metabolism, we only observed changes in glucose output and consumption. More studies are needed to explore this issue.

A strength of this study is that we selected newly diagnosed type 2 diabetes who did not receive any pharmacological treatment. Thus, we were able to exclude the potential effects of pharmacological treatment on miRNA profiles and, to some extent, reduce the potential confounding factors. In addition, we used a multidimensional “omics” approach in this population-based study to identify differentially expressed proteins associated with miR-193b-3p and to gain clues for further functional studies. Finally, we provided insight into the potential mechanism of miR-193b-3p in the development of diabetes.

There were several limitations should be considered. Firstly, miRNAs in plasma or serum are packaged in extracellular vesicles or bound to various proteins, including lipoproteins and argonaute proteins (57), and the concentration of most miRNAs in plasma are relatively low. In the present study, we screened 12 miRNAs with obvious differential expression for verification, finally four miRNAs were successfully measured and one miRNA with differential expression was validated. This might be partly due to the limited sample size in screen and validation. Secondly, we were unable to determine the source and target tissues or cells of these miRNAs. This limitation of the present study requires further research to distinguish the forms of miRNA present in plasma. Thirdly, the DFTJ cohort study was conducted in a population of middle-aged and older individuals; thus, whether these findings are applicable to other populations remains to be determined. Fourthly, in the present large study population it is difficult to perform oral glucose tolerance tests; therefore, diagnosis of diabetes based on the criteria of FPG ≥ 7.0mmol/L and/or HbA1c ≥ 6.5% may lead to misclassification. However, the positive association of miR-193b-3p with diabetes risk might not be attenuated but instead enhanced. Fifthly, “Fifthly, before testing the glucose synthesized by gluconeogenic pathway, cells were incubated in serum- and glucose-free medium for 12 h and were then incubated an additional 4 h without glucose, so that glycogen stores can be completely consumed. However, in our experimental design, we erroneously added 10% serum in the last 4 h of incubation, so we cannot atribute glucose appereance in the medium completely to gluconeogenesis, while it might be better to further study the source of increased glucose by vivo experiments or using stable isotopes. Finally, in the present study it is difficult to obtain liver tissue from diabetic patients for *in vitro* functional experiments, therefore, we investigated the effects of miR-193b-3p on glucose metabolism in HepG2 cells. However it has indicated that the HepG2 cells have relatively low similarity with human tissue (58), therefore, the functional effects of miRNA in other cell lines need to be further verified.

In conclusion, miR-193b-3p was differentially expressed in plasma of type 2 diabetes cases. With miR-193b-3p levels increased, TPI1 levels decreased both in plasma and in HepG2 cells. In addition, miR-193b-3p may affect glucose metabolism by directly targeting YWHAZ/14-3-3ζ and upregulating the FOXO1 transcription factor downstream of the PI3K-AKT pathway. Based on the results of observed in HepG2, the effects of miR-193b-3p on glucose metabolism in other tissues, such as skeletal muscle and adipose tissue, might be similar. However, it remians to be eluciated in further studies. Additionally, miR-193b-3p was verified as a plasma biomarker of diabetes, the expression of miR-193b-3p in extracellular vesicle in plasma needed test to explore the origin and destination of miR-193b-3p.

## Data Availability Statement

The datasets presented in this study can be found in online repositories. The names of the repository/repositories and accession number(s) can be found in the article/**Supplementary Material**.

## Ethics Statement

The studies involving human participants were reviewed and approved by the Ethics and Human Subject Committee of the School of Public Health, Tongji Medical College, Huazhong University of Science and Technology, and Dongfeng General Hospital, DMC. The patients/participants provided their written informed consent to participate in this study.

## Author Contributions

HH and MH conceived and designed the study. HH performed experiments, MZ, ZL, HN, JH and ZC participated in experiments. HH did the statistical analysis and drafted the manuscript. JY, HG, XZ, HY, TW, MH checked the data extraction. All authors critically revised the manuscript for important intellectual content. MH obtained fundings and supervised the study. MH has full access to all of the data and takes responsibility for the integrity of the data and the accuracy of the data analysis. All authors contributed to the article and approved the submitted version.

## Funding

This work was supported by the grants from the National Key Research and Development Program of China (2017YFC0907501 and 2016YFC0900800), the Program for HUST Academic Frontier Youth Team (2017QYTD18), and the National Natural Science Foundation (grants NSFC-81473051 and 81522040).

## Conflict of Interest

Author HY is employed by Dongfeng Central Hospital, Dongfeng Motor Corporation.

The remaining authors declare that the research was conducted in the absence of any commercial or financial relationships that could be construed as a potential conflict of interest.

## Publisher’s Note

All claims expressed in this article are solely those of the authors and do not necessarily represent those of their affiliated organizations, or those of the publisher, the editors and the reviewers. Any product that may be evaluated in this article, or claim that may be made by its manufacturer, is not guaranteed or endorsed by the publisher.
