# Supplementary material for: Plasma miR-193b-3p Is Elevated in Type 2 Diabetes and Could Impair Glucose Metabolism
Source: Front Endocrinol (Lausanne). 2022 May 27;13:814347. doi: 10.3389/fendo.2022.814347 (PMC9197112; doi:10.3389/fendo.2022.814347)
Supplement: Supplementary file 1 [file DataSheet_1.docx]

***Supplemental materials:***

**Supplemental Table 1.** Characteristics of participants in validation group (N=98 pairs)

**Supplemental Table 2.** Antibodies used in western blot

**Supplemental Table 3.** Primer sequence of miR-193b-3p target genes used in qRT-PCR

**Supplemental Table 4.** Proteins coded by miR-193b-3p target gene in three databases were identified by iTRAQ

**Supplemental Table 5.** The other 28 differentially expressed proteins identified by iTRAQ analysis.

**Supplemental Figure 1.** The work flow of the study

**Supplemental Figure 2.** Analysis of miR-193b-3p target genes

**Supplemental Figure 3.** The flow of protein selection for validation by multiple reaction- monitoring mass spectrometry (MRM-MS) from differentially expressed proteins identified by iTRAQ (isobaric tagging for relative and absolute quantification) screening

**Supplemental Figure 4.** The results of correlation analysis between miR-193b-3p and proteins in plasma. Expression level of proteins and miR-193b-3p were normalized before correlation analysis.

**Supplemental Figure 5.** Effects of miR-193b-3p on glucose metabolism in HepG2 cells**.**

**Supplemental Figure 6.** A schematic overview of the potential effect of miR-193b-3p on cellular glucose metabolism in HepG2 cells.

| **Supplemental Table 1. Characteristics of participants in validation group (N=98 pairs).** | | | |
| --- | --- | --- | --- |
| **Variables** | **DM Case** | **Controls** | ***P*** |
|  | **(n=98)** | **(n=98)** |  |
| **Male, n (%)** | 44(44.9) | 44(44.9) | 1 |
| **Age, years** | 60.8(7.0) | 60.9(7.0) | 0.919 |
| **Smoking, n (%)** | |  |  |
| Never | 66(67.3) | 69(70.4) | 0.865 |
| Current | 21(21.4) | 20(20.4) |  |
| Ever | 11(11.2) | 9(9.2) |  |
| **Drinking, n (%)** | |  |  |
| Never | 71(72.4) | 63(64.3) | 0.456 |
| Current | 23(23.5) | 29(29.6) |  |
| Ever | 4(4.1) | 6(6.1) |  |
| **Physical activity, n (%)** | 89(90.8) | 89(90.8) | 1.00 |
| **BMI, kg/m^2^** | 25.5(3.4) | 22.9(3.1) | <0.0001 |
| **WHR** | 0.89(0.06) | 0.87(0.06) | 0.002 |
| **FPG, mmol/L** | 8.9(2.5) | 5(0.4) | <0.0001 |
| **HbA1c, %** | 6.5(1.5) | 5.3(0.3) | <0.0001 |
| **TG, mmol/L** | 2.1(3.4) | 1.3(0.8) | 0.024 |
| **LDLC, mmol/L** | 2.6(0.9) | 2.9(0.8) | 0.017 |
| **HDLC, mmol/L** | 1.5(0.5) | 1.6(0.4) | 0.375 |
| **CHOL, mmol/L** | 4.9(1.3) | 4.8(1.1) | 0.667 |
| **SBP, mmHg** | 137.7(23.1) | 136.6(22.8) | 0.731 |
| **DBP, mmHg** | 80.1(13.6) | 78.9(12.7) | 0.509 |
| **Neutrophil, 10^9^/L** | 3.7(1.5) | 3.3(1.2) | 0.042 |
| **Lymphocyte, 10^9^/L** | 1.9(0.8) | 1.6(0.5) | 0.021 |
| **Monocyte, 10^9^/L** | 0.4(0.2) | 0.3(0.2) | 0.097 |
| **Eosnophils, 10^9^/L** | 0.2(0.2) | 0.1(0.1) | 0.036 |
| **Basophil, 10^9^/L** | 0.2(0.2) | 0.1(0.2) | 0.588 |
| **WBC, 10^9^/L** | 5.9(1.8) | 5.3(1.4) | 0.01 |
| **RBC, 10^12^/L** | 4.6(0.5) | 4.5(0.5) | 0.523 |
| **PLT, 10^9^/L** | 184.6(51.8) | 197.3(52.4) | 0.091 |
| **Family history of diabetes, n (%)** | 7(7.2) | 7(7.1) | 1.00 |
| Note: WHR=waist-to-hip ratio | | | |

| **Supplemental Table 2. Antibodies used in western blot** | | |
| --- | --- | --- |
| **Antibodies** | **Item No** | **Manufacturer** |
| TPI1 | 10713-1-AP | Proteintech |
| KRAS | 12063-1-AP | Proteintech |
| SOS2 | AF6260-SP | Novus Biologicals |
| YWHAZ | ab133323 | Abcam |
| IR | 3025 | Cell Signaling |
| GLUT2 | ab192599 | Abcam |
| FOXO1 | 18592-1-AP | Proteintech |
| PCK1 | 16754-1-AP | Proteintech |
| G6PC | 22169-1-AP | Proteintech |
| GSK3A | 22104-1-AP | Proteintech |
| GYS2 | 10566-1-AP | Proteintech |
| Tublin | 66031-1-Ig | Proteintech |
| β-actin | 60008-1-Ig | Proteintech |

| **Supplemental Table 3. Primer sequence of miR-193b-3p target genes used in qRT-PCR** | | |
| --- | --- | --- |
| **Gene** | **Forward primer sequence** | **Reverse primer sequence** |
| *GLUT2* | ACTCAACCAGCATTTTTCAGAC | CACAAACAAACATCCCACTCAT |
| *IR* | TCCGTGGAGGATAATTACATCG | AGTGACTATGAGTCCAACATCG |
| *TPI* | GCATCACTGAGAAGGTTGTTTT | AGAGCCTCCATAAATGATACGG |
| *YWHAZ* | CTCTCTTGCAAAGACAGCTTTT | GTCCACAATGTCAAGTTGTCTC |
| *SOS2* | GTCAGAACTGAAATCGCAGAAG | AAAGAAAGGCTTCTCGAAACAC |
| *KRAS* | CGACACAGCAGGTCAAGAGG | GGCATCATCAACACCCTGTCT |
| *FOXO1* | AAACACCAGTTTGAATTCACCC | TCGACTTATTGTCCTGAAGTGT |
| *PCK1* | GGTTCCCAGGGTGCATGAAA | CACGTAGGGTGAATCCGTCAG |
| *G6PC* | TCTACGTCCTCTTCCCCATC | TCAGTATCCAAAACCCACCAG |
| *GYS2* | GAGTGAGGACGAGGAGGATC | CTCTGGTGCACGGATGTT |
| *GSK3A* | AGGAGAACCCAATGTTTCGTAT | ATCCCCTGGAAATATTGGTTGT |
| *β-actin* | TGCTCCTCCTGAGCGCAAGTA | CCACATCTGCTGGAAGGTGGA |

| **Supplemental Table 4. Proteins coded by miR-193b-3p target gene in three databases were identified by iTRAQ** | | |
| --- | --- | --- |
| **Database** | **Target Gene** | **Proteins** |
| TargetScan | *C1QC, KIT, PSMA5, YWHAZ, DYNLL2, MYLK, HEG1* | P02747, A0A0U2N547, P28066, P63104, Q96FJ2, Q15746, Q9ULI3 |
| miRDB | *C1QC, KIT, PSMA5, YWHAZ, SVEP1, ARPC5* | P02747, A0A0U2N547, P28066, P63104, Q4LDE5, O15511 |
| miRTarBase | *KIT, YWHAZ, TPI1, TLN1, COTL1, PFN1, C5, UGP2, ENDOD1, MEGF8, CFL1, ACTN1, YWHAQ, HPRT1, GXYLT1, LAMB1, ACTN4, PTPRG, PGAM1, FLNA, ZYX, MYH9, HSPA1A, GSS, GDI2, MYLK, CDH1, PTPRF, TPM2* | A0A0U2N547, P63104, P60174, Q9Y490, Q14019, P07737, P01031, A0A140VKE1, O94919, Q7Z7M0, P23528, P12814, P27348, P00492, Q4G148, P07942, O43707, P23470, P18669, P21333, Q15942, P35579, P0DMV9, P48637, P50395, Q15746, B3GN61, P10586, Q5TCU3 |
| Note: iTRAQ=isobaric tags for relative and absolute quantification; Proteins were listed by uniport accession. | | |

| **Supplemental Table 5. The other 28 differentially expressed proteins identified by iTRAQ analysis.** | | | | |
| --- | --- | --- | --- | --- |
| **Accession** | **Description** | **Peptides Coverage** | **Case vs. Control** | |
|  |  |  | **Fold change** | ***P* value** |
| Q4G148 | Glucoside xylosyltransferase 1 (GXYLT1) | 1.82 | 1.3 | 0.154 |
| B3GN61 | Truncated E-cadherin (CDH1) | 6.33 | 1.12 | 0.023 |
| A0A0U2N547 | Mast/stem cell growth factor receptor Kit isoform 3 (KIT) | 4.02 | 1.1 | 0.237 |
| P35579 | Myosin-9 (MYH9) | 27.14 | 1.08 | 0.444 |
| P01031 | Complement C5 (C5) | 41.35 | 1.05 | 0.222 |
| Q9ULI3 | Protein HEG homolog 1 (HEG1) | 7.31 | 1.01 | 0.847 |
| P23470 | Receptor-type tyrosine-protein phosphatase gamma (RTPRG) | 4.5 | 1.01 | 0.885 |
| P07942 | Laminin subunit beta-1 (LAMB1) | 3.08 | 1.01 | 0.909 |
| P02747 | Complement C1q subcomponent subunit C (C1QC) | 21.22 | 1 | 0.967 |
| Q96FJ2 | Dynein light chain 2, cytoplasmic (DYNLL2) | 7.87 | 1 | 0.989 |
| P28066 | Proteasome subunit alpha type-5 (PSMA5) | 22.41 | 0.97 | 0.783 |
| A0A140VKE1 | Testis tissue sperm-binding protein Li 58p (UGP2) | 12.88 | 0.92 | 0.45 |
| P23528 | Cofilin-1 (CFL1) | 60.24 | 0.91 | 0.327 |
| P50395 | Rab GDP dissociation inhibitor beta (GDI2) | 32.81 | 0.91 | 0.087 |
| P18669 | Phosphoglycerate mutase 1 (PGAM1) | 25.2 | 0.9 | 0.276 |
| Q15746 | Myosin light chain kinase, smooth muscle (MYLK) | 4.18 | 0.9 | 0.274 |
| Q5TCU3 | Tropomyosin beta chain (TPM2) | 49.65 | 0.88 | 0.209 |
| Q15942 | Zyxin (ZYX) | 29.9 | 0.87 | 0.152 |
| P0DMV9 | Heat shock 70 kDa protein 1B (HSPA1A) | 28.08 | 0.87 | 0.035 |
| P63104 | 14-3-3 protein zeta/delta (YWHAZ) | 60 | 0.86 | 0.059 |
| P21333 | Filamin-A (FLNA) | 33.62 | 0.86 | 0.021 |
| O43707 | Alpha-actinin-4 (ACTN4) | 32.38 | 0.86 | 0.059 |
| P00492 | Hypoxanthine-guanine phosphoribosyltransferase (HPRT1) | 13.76 | 0.84 | 0.279 |
| P48637 | Glutathione synthetase (GSS) | 8.23 | 0.83 | 0.173 |
| P12814 | Alpha-actinin-1 (ACTN1) | 50.45 | 0.82 | 0.066 |
| P10586 | Receptor-type tyrosine-protein phosphatase F (PTPRF) | 5.19 | 0.82 | 0.071 |
| P27348 | 14-3-3 protein theta (YWHAQ) | 44.49 | 0.79 | 0.168 |
| O94919 | Endonuclease domain-containing 1 protein ( ENDOD1) | 10 | 0.77 | 0.149 |
| Notes: Accession was the number of proteins in Uniprot database .Peptides coverage: The number of amino acids in the peptide detected by mass spectrometry accounted for a proportion of the total number of amino acids in the protein. | | | | |

|  |
| --- |

**
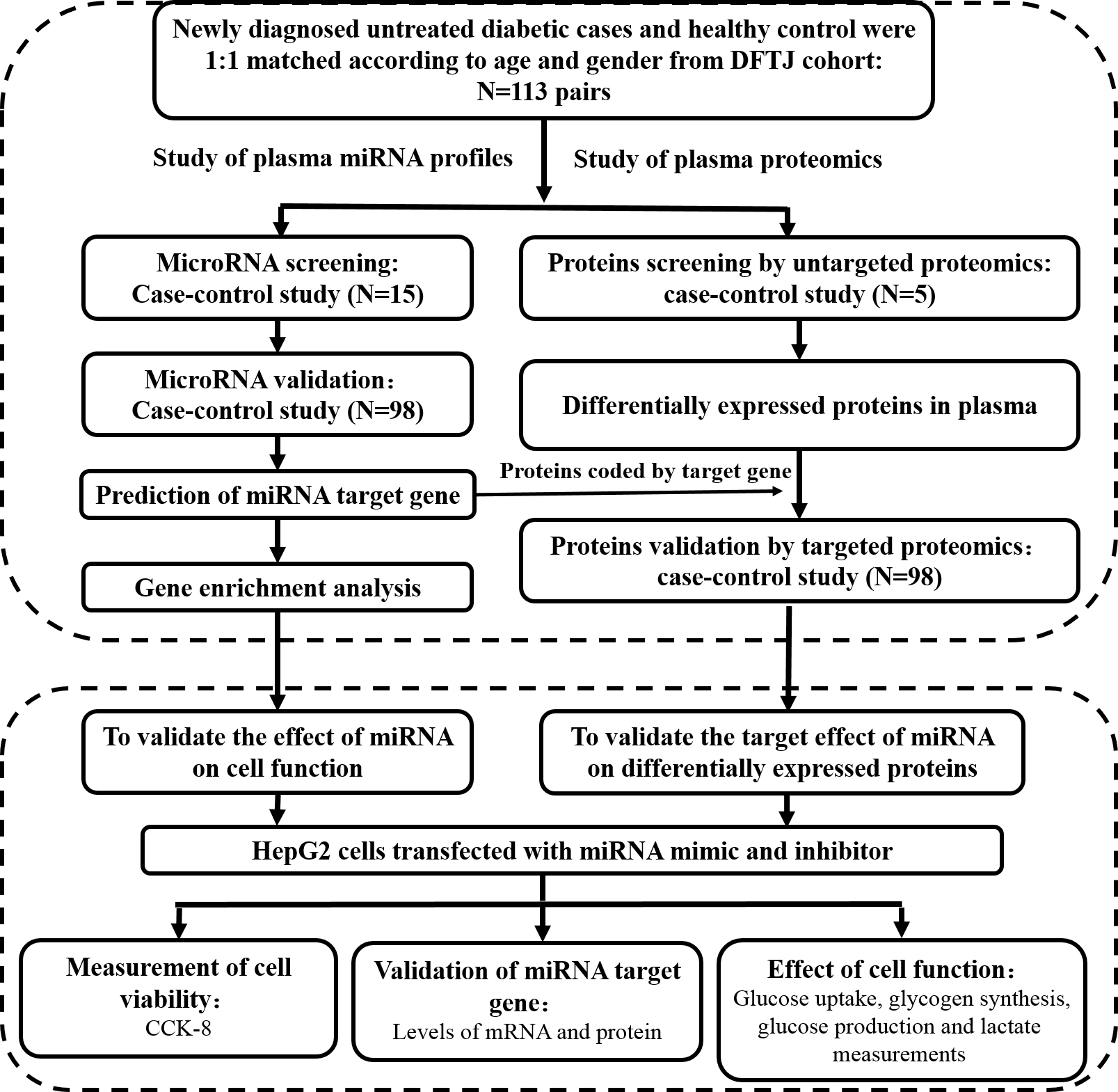
**

**Supplemental Figure 1. The work flow of the study.**

**
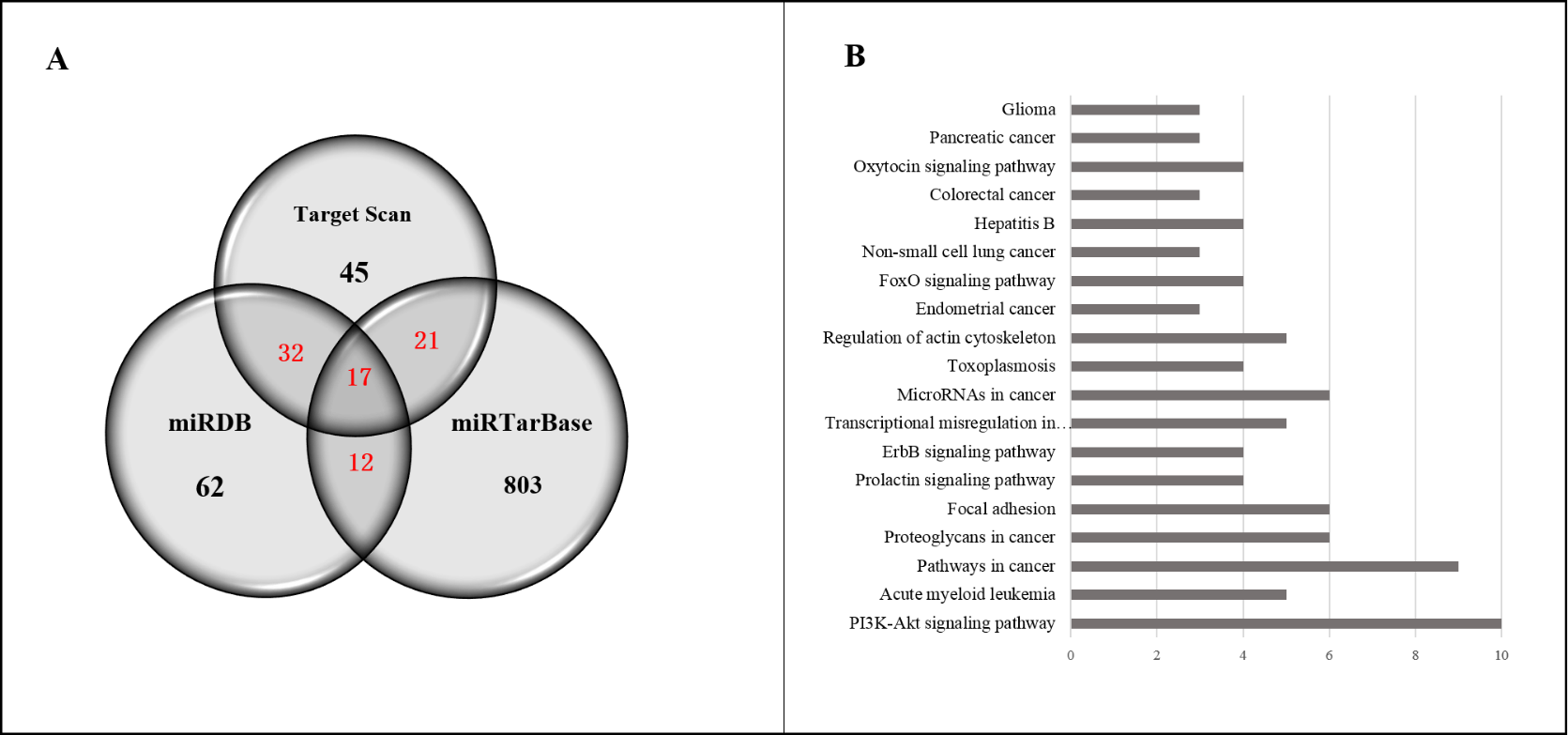
**

**Supplemental Figure 2. Analysis of miR-193b-3p target genes.**

(A) The Venn diagram of genes predicted by three databases (miRDB, miRTarBase, and TargetScan). (B) The pathways of miR-193b-3p target genes. The pathway enrichment of the target genes was analyzed by Kyoto Encyclopedia of Genes and Genomes (KEGG) (http://www.genome. jp/kegg/). The bioinformatics analysis pathway enrichment was performed by the DAVID tools (http://david.abcc.ncifcrf.gov/). Genes predicted by at least two databases were enrolled to pathway enrichment analysis.

**
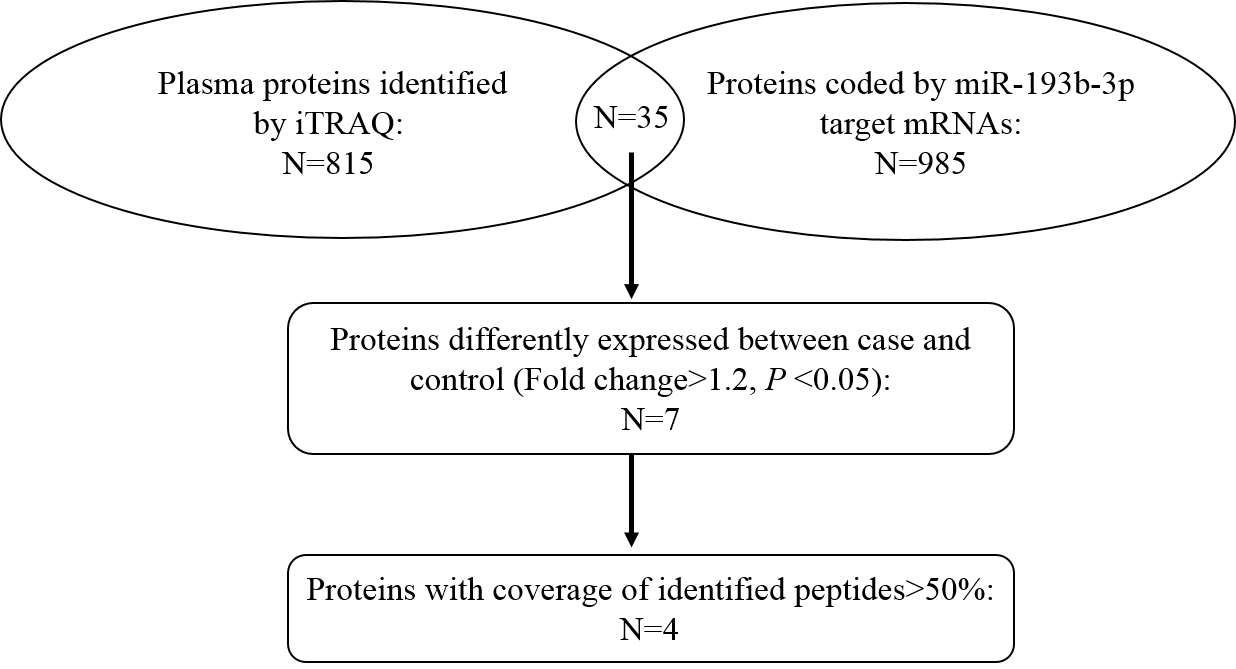
**

**Supplemental Figure 3. The flow of protein selection for validation by MRM-MS from differentially expressed proteins identified by iTRAQ**

Target sites of miRNA on mRNA were predict by miRTarBase (<http://mirtarbase.mbc.nctu.edu.tw/php/index.php>), TargetScan (<http://www.targetscan.org/>), and miRDB (http://mirdb.org/), Proteins coded by target mRNAs were analyzed in Uniprot Database (https://www.uniprot.org/). Peptides coverage: The number of amino acids in the peptide detected by mass spectrometry accounted for a proportion of the total number of amino acids in the protein.


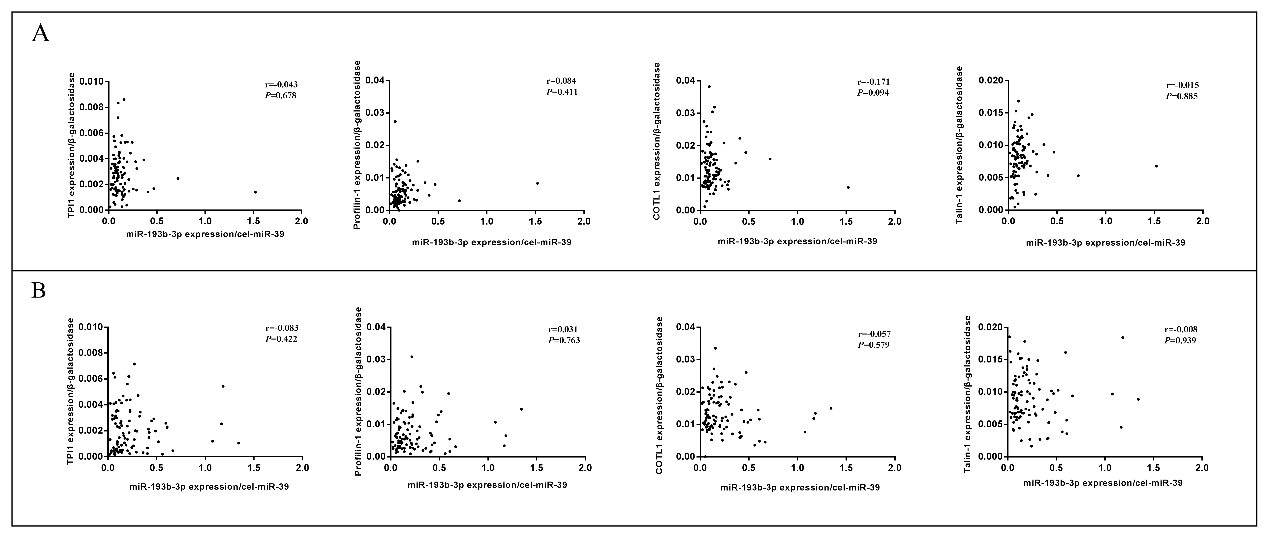


**Supplemental Figure 4. The results of correlation analysis between miR-193b-3p and proteins in plasma.**

Expression level of proteins and miR-193b-3p were normalized before correlation analysis. Panel A indicates correlation between miR-193b-3p and proteins in plasma in cases, panel B indicates correlation between miR-193b-3p and proteins in plasma in controls.

**
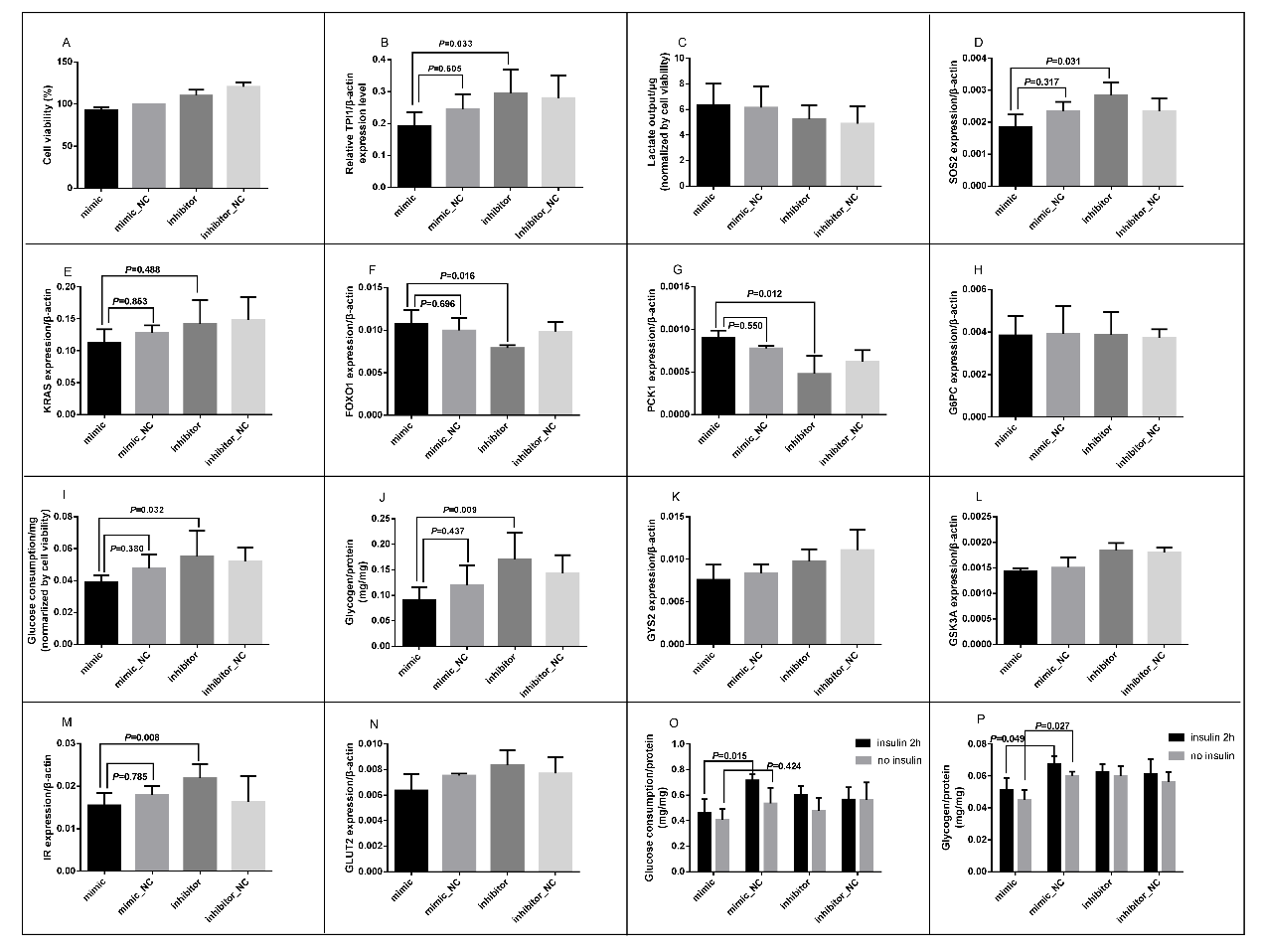
**

**Supplemental Figure 5. Effects of miR-193b-3p on glucose metabolism in HepG2 cells.**

Mimic: HepG2 cells transfected with miR-193b-3p mimic; mimic_NC: cells transfected with negative control of mimic; inhibitor: cells transfected with miR-193b-3p inhibitor; inhibitor_NC: cells transfected with negative control of inhibitor. (A) The cell viability was assessed by Cell Counting Kit-8 assay, Y-axis represent the OD value measured by multiscan spectrum after incubated in medium with CCK8. Bar graphs shows the level of mRNA: (B) TPI1; (D) SOS2; (E) KRAS; (F) FOXO1; (G) PCK1; (H) G6PC; (K) GYS2; (L) GSK3A; (M) IR; (N) GLUT2. (C) The extracellular lactate levels. (I) The glucose consumption after transfection. (J) The level of intracellular glycogen after transfection. (O) The glucose consumption after transfection and incubation in the presence or absence of insulin. (P) The level of intracellular glycogen after transfection and incubation in the presence or absence of insulin. Bar graphs marked without *P* value indicate no difference exist among 4 groups: (A), (C), (H), (K), (L), (N)

**
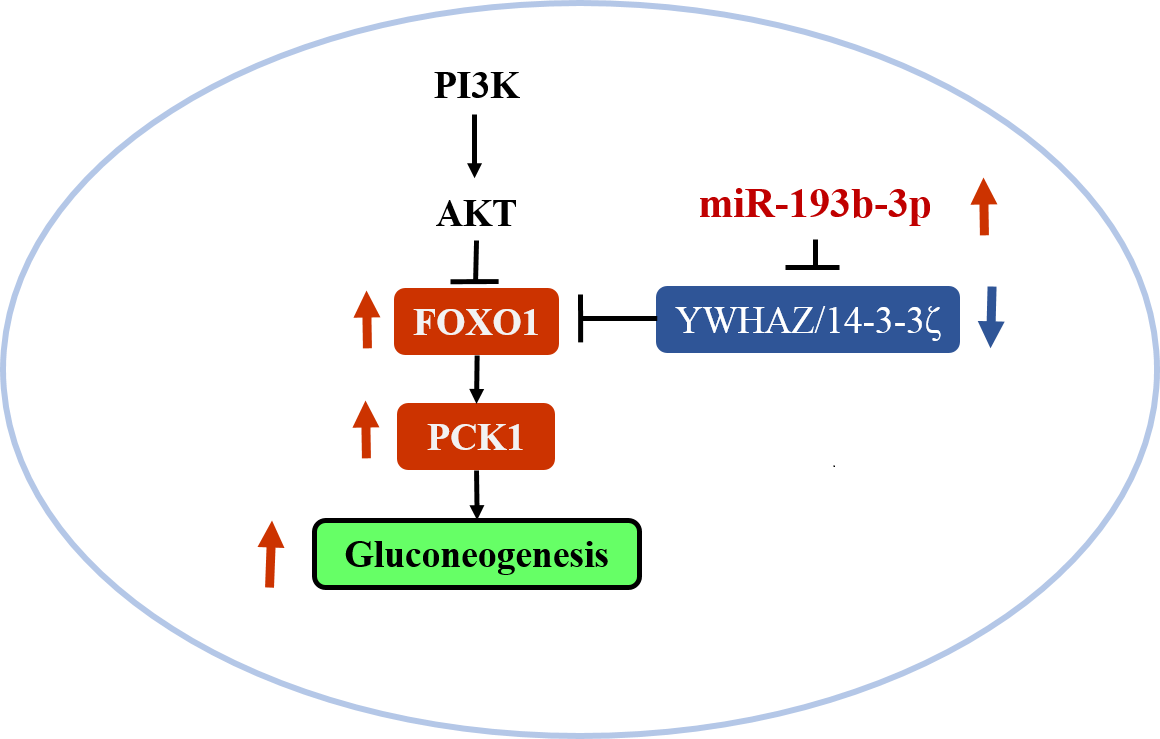
**

**Supplemental Figure 6. A schematic overview of the potential effect of miR-193b-3p on cellular glucose metabolism in HepG2 cells.**

miR-193b-3p can target to YWHAZ/14-3-3ζ, and subsequently up-regulate the transcription factor FOXO1 in the downstream of PI3K-AKT pathway, then FOXO1 increases the expression of PCK1 and enhances gluconeogenesis. Red color indicates that the protein levels increased, blue color indicates that the protein levels decreased, and green color indicates metabolic endpoints.
